# Soluble mucus component CLCA1 modulates expression of leukotactic cytokines and BPIFA1 in murine alveolar macrophages but not in bone marrow-derived macrophages

**DOI:** 10.1007/s00418-018-1664-y

**Published:** 2018-04-02

**Authors:** Nancy A. Erickson, Kristina Dietert, Jana Enders, Rainer Glauben, Geraldine Nouailles, Achim D. Gruber, Lars Mundhenk

**Affiliations:** 1 Department of Veterinary Pathology, Freie Universität Berlin, Robert-von-Ostertag-Strasse 15, 14163 Berlin, Germany; 2 Division of Gastroenterology, Infectiology and Rheumatology, Medical Department, Charité—Universitätsmedizin Berlin, Hindenburgdamm 30, 12200 Berlin, Germany; 3 Department of Infectious Diseases and Pulmonary Medicine, Charité—Universitätsmedizin Berlin, Charitéplatz 1, 10117 Berlin, Germany

**Keywords:** SPLUNC, Pneumonia, Animal model, Translatability, mCLCA3, Gob-5

## Abstract

The secreted airway mucus cell protein chloride channel regulator, calcium-activated 1, CLCA1, plays a role in inflammatory respiratory diseases via as yet unidentified pathways. For example, deficiency of CLCA1 in a mouse model of acute pneumonia resulted in reduced cytokine expression with less leukocyte recruitment and the human CLCA1 was shown to be capable of activating macrophages in vitro. Translation of experimental data between human and mouse models has proven problematic due to several CLCA species-specific differences. We therefore characterized activation of macrophages by CLCA1 in detail in solely murine ex vivo and in vitro models. Only alveolar but not bone marrow-derived macrophages freshly isolated from C57BL6/J mice increased their expression levels of several pro-inflammatory and leukotactic cytokines upon CLCA1 stimulation. Among the most strongly regulated genes, we identified the host-protective and immunomodulatory airway mucus component BPIFA1, previously unknown to be expressed by airway macrophages. Furthermore, evidence from an in vivo *Staphylococcus aureus* pneumonia mouse model suggests that CLCA1 may also modify BPIFA1 expression in airway epithelial cells. Our data underscore and specify the role of mouse CLCA1 in inflammatory airway disease to activate airway macrophages. In addition to its ability to upregulate cytokine expression which explains previous observations in the *Clca1*-deficient *S. aureus* pneumonia mouse model, modulation of BPIFA1 expression expands the role of CLCA1 in airway disease to involvement in more complex downstream pathways, possibly including liquid homeostasis, airway protection, and antimicrobial defense.

**Electronic supplementary material:**

The online version of this article (10.1007/s00418-018-1664-y) contains supplementary material, which is available to authorized users.

## Introduction

Chloride channel regulator, calcium-activated 1, CLCA1, is selectively expressed by goblet and other mucin-producing cells and is secreted into the mucus layer of airways, the intestinal tract and other mucosal linings in man and mice (Gibson et al. 2005; Gruber et al. 1998; Leverkoehne and Gruber 2002). The originally confusing nomenclature of murine CLCA1, previously termed mCLCA3 or goblet cell protein-5 (gob-5), was harmonized with respect to the human and rat nomenclature in accordance with the Human Gene Nomenclature Committee and the Rat Genome Database (Erickson et al. 2015). The soluble heterodimer consists of two posttranslational cleavage products, a 75 kDa amino- and a 35 kDa carboxy-terminal protein, that are processed and glycosylated from a primary 125 kDa translation product (Mundhenk et al. 2006). CLCA1 has repeatedly been hypothesized to play a modulatory role in chronic respiratory diseases such as asthma, cystic fibrosis (CF), and chronic obstructive pulmonary disease (Hegab et al. 2004; Kamada et al. 2004; Patel et al. 2009). In particular, inflammatory conditions have consistently been associated with increased CLCA1 expression in airway epithelial cells and its expression by far exceeded that of other mediators of inflammation (Hauber et al. 2010; Zhou et al. 2001). High amounts of CLCA1 were present in the bronchoalveolar lavage fluid (BALF) of asthmatic patients (Gibson et al. 2005). In an ovalbumin-induced mouse model of asthma, the murine CLCA1 ortholog was strongly secreted into the airway fluids (Gibson et al. 2005). Furthermore, asthmatic mice treated with anti-CLCA1 antibodies showed a marked reduction of airway inflammation (Song et al. 2013). It has thus been suggested that CLCA1 may also serve as a diagnostic marker as well as a potential therapeutic target for inflammatory airway diseases (Patel et al. 2009). However, its exact function in the complex pathways of airway inflammation has not yet been established (Patel et al. 2009) and partially contradictory results have been obtained in humans and mice.

Substantial genomic, structural, functional, and expressional differences have been described for several orthologous CLCA family members from different species. For example, *Clca3* possesses one functional gene copy in several mammalian species but two distinct functional copies in cattle, whereas in humans and in pigs it is a non-functional pseudogene (Gruber and Pauli 1999; Mundhenk et al. 2018; Plog et al. 2009). Interestingly, in the mouse, two gene duplication events resulted in three apparently functional CLCA3 proteins which are expressed in different cellular niches (Mundhenk et al. 2018; Patel et al. 2009). The *Clca4* gene is duplicated in the pig and the mouse with two or three, respectively, apparently distinctly regulated proteins, expressed in different cell types and functional niches (Patel et al. 2009; Plog et al. 2015). Other mammals appear to possess only a single CLCA4 protein (Plog et al. 2015). While such interspecies variations seem to be absent from CLCA1 on the genomic level, several functional differences have been described between human and mouse CLCA1 proteins. The human CLCA1 has been established as a key regulator of mucus cell metaplasia in inflammatory airway disease via interleukin (IL)-13-driven mucus gene transcription (Alevy et al. 2012). By contrast, a related mouse model failed to mirror the human data with regard to IL-13-dependence of CLCA1-mediated airway mucus production (Alevy et al. 2012). Moreover, CLCA1 modulates activation of a transmembrane protein 16A (TMEM16A, anoctamin-1)-mediated calcium-dependent chloride current (CaCC) in a paracrine and self-cleavage-dependent fashion (Sala-Rabanal et al. 2015). This non-cystic fibrosis transmembrane conductance regulator (CFTR)-mediated, alternative chloride current has been discussed as an important modulator of disease and therapeutic target in CF patients (Berschneider et al. 1988; Bronsveld et al. 2000; Taylor et al. 1988; Willumsen and Boucher 1989). However, tracheal instillation of IL-13 in murine airways resulted in overexpression of CLCA1 but failed to induce CaCC activity (Mundhenk et al. 2012). Moreover, CaCC was unchanged in the airways of *Clca1*-deficient (*Clca1*^−/−^) mice. It therefore seems that, in contrast to human airways, CLCA1 may not play a role in CaCC-mediated chloride secretion in the mouse (Mundhenk et al. 2012).

More recently, a third functional role has been identified for CLCA1 in that it seems to act on macrophages as signaling molecule, thereby modulating inflammatory responses. Specifically, the human CLCA1 induced a pro-inflammatory cytokine response including IL-8, the human ortholog to the murine chemokine (C-X-C motif) ligands Cxcl-1 (alias KC, keratinocyte chemoattractant) and Cxcl-2 (alias MIP-2α, macrophage inflammatory protein 2-alpha), as well as IL-6, IL-1β and tumor necrosis factor (TNF)-α in the human monocyte cell line U-937 which had been artificially differentiated into airway macrophage-like cells (Ching et al. 2013). Similar effects were observed when human CLCA1 was added to primary porcine alveolar macrophages (Ching et al. 2013). Whether the same pathways are in effect in the mouse remains to be elucidated, in particular due to numerous established differences between murine and human immune functions. In the mouse, only circumstantial evidence has so far pointed toward a role for CLCA1 in early airway inflammation. Here, expression levels of specific cytokines in whole tissue lysates and leukocyte recruitment to airways were affected in in vivo *Clca1*^−/−^ models with contradictory results depending on the stimuli used (Dietert et al. 2014; Long et al. 2006). Specifically, *Clca1* deficiency resulted in decreased *Cxcl*-*1* and *Cxcl-2* responses with decreased neutrophil recruitment and reduced expression of the pro-inflammatory cytokine *Il-17A* in a mouse model of acute *Staphylococcus* (*S*.) *aureus* pneumonia (Dietert et al. 2014). By contrast, increased neutrophil recruitment preceded by CXCL-1 upregulation was seen following intranasal lipopolysaccharide (LPS) challenge in *Clca1*^−/−^ mice (Long et al. 2006). Clearly, the exact mechanisms of how CLCA1 may execute such effects remain to be established with careful consideration of possible differences between humans and mice also in this regard.

Here, we investigated the direct effects of murine CLCA1 action on cytokine expression levels in freshly ex vivo-derived macrophages in a solely murine model. Alveolar and bone marrow-derived macrophages (BMDM) isolated from C57BL/6J mice were stimulated with CLCA1. Several pro-inflammatory cytokines and chemokines were induced on the mRNA and protein levels in alveolar but not bone marrow-derived macrophages. Using global gene expression analyses, we further identified other genes that were differentially regulated in alveolar macrophages upon activation by CLCA1, including the host-protective and immunomodulatory airway mucus component BPI fold containing family A member 1 (*Bpifa1*), formerly known as short palate, lung, and nasal epithelium clone (PLUNC) 1 (SPLUNC1) protein (Britto and Cohn 2015). The results support the hypothesis of murine CLCA1 directly and specifically activating complex alveolar macrophage signaling and broaden the spectrum of downstream pathways by modulation of the multifunctional BPIFA1.

## Materials and methods

### Cell culture, transfection, and supernatant collection

All cell cultures were grown at 37 °C in a humidified atmosphere with 5% CO_2_. Human embryonic kidney cells (HEK293) were grown in very low endotoxin Dulbecco’s MEM medium (VLE DMEM; Biochrom GmbH, Berlin, Germany) supplemented with 10% heat-inactivated fetal bovine serum (FBS; B&S FCS Gold Plus chromatographiert, Bio & Sell, Feucht, Germany). Based on Ching et al. (Ching et al. 2013), mouse CLCA1 protein was generated as follows. Briefly, HEK 293 cells were seeded in 10-cm plates, grown for 24 h to 80% confluence and transfected with the murine wild-type (WT) CLCA1 (Leverkoehne and Gruber 2002) or mock-transfected with the pcDNA3.1 + vector alone (pcDNA; Life technologies, Darmstadt, Germany). Transfections were performed using the Turbofect transfection reagent (Thermo Scientific, Darmstadt, Germany) according to the manufacturer’s protocol. 24 h after transfection, medium was replaced by 5 ml FBS-free VLE DMEM and incubated for 6 h, collected, centrifuged at 500×*g* for 5 min at 4 °C to remove remaining cells. Macromolecules were concentrated by centrifuging 10 ml of the collected media at 3000×*g* and 4 °C through Vivaspin 15R columns of 5000 MWCO (Sartorius Stedim Biotech GmbH, Goettingen, Germany) to 100 µl of conditioned medium (CM). All cell culture, transfection, supernatant collection, and Vivaspin concentration steps of CLCA1- and pcDNA-mock-transfected cell culture supernatants were performed identically. Hence, the pcDNA-CM contained the same amount of any secreted protein other than CLCA1. For macrophage stimulation, 50 or 100 µl of CLCA1-CM or 100 µl of pcDNA-CM as negative control was applied. Total protein concentrations were determined with the Micro BCA™ Protein Assay Kit (Thermo Scientific, Rockford, IL, USA).

CLCA1 was immunoprecipitated using the CLCA1-specific antibody α-mCLCA3-C-1p (Bothe et al. 2011) followed by sodium dodecyl sulfate polyacrylamide gel electrophoresis (SDS-PAGE). Specificity of this antibody was determined via Western Blot using α-p3b2 antibody as described earlier (Bothe et al. 2011), shown in Online Resource 1a (Electronic Supplementary Material). The precipitated CLCA1 protein was also visualized on a Coomassie-stained SDS-PAGE gel compared to the pcDNA control, representatively shown in Online Resource 1b (Electronic Supplementary Material). Protein sizes were estimated using the Spectra™ Multicolor Broad Range Protein Ladder (Thermo Scientific, Darmstadt, Germany). For a graphical illustration of the experimental setup, see Online Resource 2 (Electronic Supplementary Material).

### Isolation of murine alveolar macrophages

**Fig. 1:**
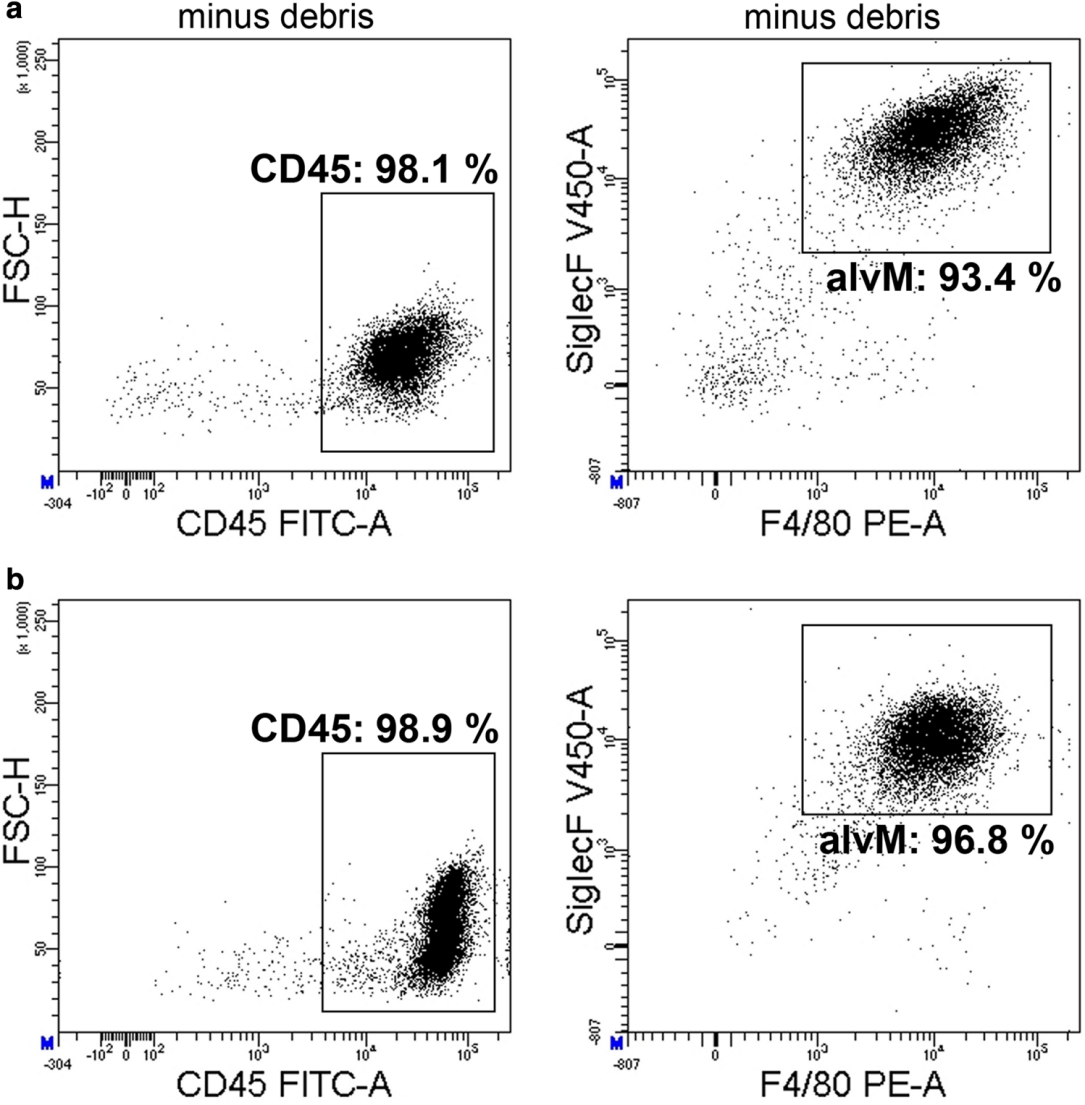
Phenotyping of BAL-derived cell culture identified over 95% murine alveolar macrophages. Representative dot blots showing leukocytes (CD45+) and alveolar macrophage (alvM) (Siglec-F + F4/80+) proportions (%) of the BAL of WT mice **a** pre- and **b** 24 h post-seeding. Cellular debris was excluded by side scatter (SSC-A) and forward scatter (FSC-A) gating. Frequencies of CD45 + leukocytes (left) and Siglec-F + F4/80 + alveolar macrophages (right) among all events minus debris depicted in blots. The BAL consisted of 93.4% alveolar macrophages pre-seeding and 96.8% post-seeding

WT and *Clca1*^−/−^ mice on a C57BL/6J background (Patel et al. 2006) 8–12 weeks of age were anesthetized by intraperitoneal injection of premixed ketamine (3.2 mg) and xylazine (1.5 mg), heparinized and killed by exsanguination via the caudal vena cava. Immediately thereafter, the mice were tracheotomized and bronchioalveolar lavage (BAL) was performed six times with 800 µl pre-warmed phosphate-buffered saline (PBS; Biowest, Nuaillé, France) supplemented with 2 mM ethylenediaminetetraacetic acid (EDTA; Merck, Darmstadt, Germany). BALF from two mice were pooled for one single experimental data point. BALF was centrifuged at 500×*g* for 5 min at 4 °C. The resulting pellet was resuspended in 400 µl, complete medium, i.e., VLE RPMI 1640 Medium (VLE RPMI; Biochrom AG, Berlin, Germany), supplemented with 1% penicillin–streptomycin (Biowest, Nuaillé, France) and 10% FBS. 5 × 10^5^ cells were seeded into each well of a 24-well plate. Medium was replaced after 2 h and cells were incubated for 24 h prior to stimulation (Kostadinova et al. 2016). To test the purity of the alveolar macrophage cell cultures, cells of the BAL were phenotyped via flow cytometry (Chavez-Santoscoy et al. 2012; Zhang et al. 2008) pre-seeding and 24 h post-seeding. For measurements at 24 h post-seeding, alveolar macrophages were detached from tissue culture plates by removal of the media and replacement with ice-cold 5 mM EDTA in 1× PBS. Tissue culture plates were placed on ice for 30 min and adherent macrophages were removed from wells by gentle scraping with a cell scraper. Pre-seeded and post-seeded cells were pre-incubated with blocking antibody (anti-CD16/32) and stained with anti-CD45 (30-F11), anti-Siglec-F (E50-2440, all BD Biosciences, Heidelberg, Germany), and anti-F4/80 (BM8, eBioscience, Frankfurt/Main, Germany). All stained cells were acquired using a BD FACS Canto II and analyzed with BD FACSDiva software. 93.4 and 96.8% of the BAL-derived cells were identified as alveolar macrophage pre-seeding or 24 h post-seeding, respectively (Fig. 1).

### Isolation and differentiation of murine BMDM

BMDM were isolated from WT mice by flushing a femoral bone with 10 ml of sterile VLE RPMI supplemented with 1% penicillin–streptomycin using a sterile cannula. The obtained cell suspension was centrifuged at 500×*g* for 5 min at 4 °C and resuspended in macrophage differentiating medium containing 10 ml VLE RPMI supplemented with 1% non-essential amino acids (NEA; 100×; Biochrom AG, Berlin, Germany), 1% HEPES-Buffer (1 M; Biochrom AG), 1% sodium pyruvate (100 mM; Biochrom AG), 10% L929-CM, 10% FBS and 1% penicillin–streptomycin. 5 × 10^5^ cells were seeded into each well of a 24-well plate. After 72 h, 100 µl of differentiating medium was added to each well. Cells were allowed to differentiate for 6 days (Naujoks et al. 2016). Identically to alveolar macrophages, medium was replaced by 400 µl of complete medium 24 h prior to stimulation.

### Stimulation of alveolar macrophages and activation of BMDM

Alveolar and bone marrow-derived macrophages (*n* = 3–5) were incubated with 50 or 100 µl of WT CLCA1− or 100 µl of pcDNA-CM in 500 µl total volume for 24 h. *Clca1*^−/−^ alveolar macrophages were stimulated with 100 µl of CLCA1-CM only to reduce the number of animals used for the study. As a positive control, macrophages were incubated with 100 µl of pcDNA-CM supplemented with lipoteichoic acid, LTA, which activates macrophages through toll-like receptor 2, TLR2 (Schroder et al. 2003) or LPS as an exclusive TLR4 agonist (Beutler et al. 2001). Alveolar macrophages were incubated with 20 ng and BMDM with 200 ng LTA (Invivogen, Toulouse, France) per ml pcDNA medium, respectively. Alveolar macrophages were incubated with 10 ng and BMDM with 100 ng of LPS (Enzo Life Sciences GmbH, Lörrach, Germany) per ml pcDNA medium, respectively.

### Trachea and lung tissues from an acute *S. aureus* pneumonia mouse model

Trachea and lung mRNA or tissues had been obtained from female *Clca1*^−/−^ and WT mice, both C57BL/6J, after transnasal infection with *S. aureus* Newman in 20 ml sterile PBS for subsequent reverse transcriptase quantitative polymerase chain reaction (RT-qPCR) analyses or immunohistochemical analyses, respectively. Controls had received 20 ml of sterile PBS (Dietert et al. 2014).

### RNA isolation and RT-qPCR

Total macrophage RNA was isolated using the NucleoSpin® RNA XS isolation Kit (Macherey-Nagel, Düren, Germany) according to the manufacturer’s instructions. Trachea or lung parenchyma-derived poly-A + mRNA from a previous study using a murine acute *S. aureus* pneumonia infection model was reverse transcribed as described (Dietert et al. 2014). Primer and probe design, RT-qPCR protocols and data analyses were performed as described (Dietert et al. 2014).

Macrophage transcript expression levels of *Cxcl-1, Cxcl-2, Il-1β, Il-6, Il-17A, Tnf-α*, *Bpifa,* and *Ccl5* were determined and normalized to the internal reference genes glyceraldehyde-3-phosphate dehydrogenase (*Gapdh*), elongation factor 1α (*Ef-1α*) and ß-2 microglobulin (*B2m*) as previously described (Dietert et al. 2014). *Bpifa1* transcript expression levels from tracheal and lung tissue from the *S. aureus* pneumonia model were determined and also normalized to the internal reference genes. Primers and probes for *Gapdh, Il-1β, Il-17A* (Giulietti et al. 2001), *Ef-1α* (Braun et al. 2010), *B2m* (Norris et al. 2000), *Cxcl-1* and -*2* (Dietert et al. 2014), *Bpifa1* (Liu et al. 2013a), *Tnf-α* (Innamorato et al. 2008), *Il-6* (Bloks 2009), and primers for CC-chemokine ligand 5 (*Ccl5*) (Ishida et al. 2012) were used as described. The probe for *Ccl5* was designed using Primer3 software (WWW primertool, Whitehead Institute of Biomedical Research). Primer and probe sequences are listed in Online Resource 3 (Electronic Supplementary Material).

### Cytometric bead array and multiplex assay

Cell culture supernatants of alveolar or bone marrow-derived macrophages were collected and centrifuged at 500×*g* for 5 min at 4 °C. The cytokines CXCL-1, TNF-α, IL-1β, IL-6, and IL-17A were quantified in the supernatant via cytometric bead array (CBA) using a FACSCantoII and the FACSDiva software (all BD Biosciences) as described (Batra et al. 2012; Glauben et al. 2006) or using a cytokine protein multiplex assay (Bioplex, Bio-Rad, Hercules, CA) according to the manufacturer’s instructions. To exclude the introduction of cytokine expression with CM, murine cytokines which were detected after stimulation of alveolar macrophages with CLCA1 were measured in the CM of pcDNA- or CLCA1-transfected HEK293 cells. As the transfected cell line is of human origin, a human CBA assay was performed in addition to the murine assay. No induction by CLCA1 of any of the measured cytokines was observed, shown in the Online Resource 4 (Electronic Supplementary Material).

### Immunohistochemistry

Formalin-fixed, paraffin-embedded trachea and lung tissue samples from the previous acute *S. aureus* mouse pneumonia study (Dietert et al. 2014) were cut at 2 µm thickness and mounted on adhesive glass slides. After dewaxing in xylene and rehydration in decreasing ethanol concentrations, antigen retrieval was performed by microwave heating (600 W) in 10 mM citric acid (750 ml, pH 6.0) for 12 min. Slides were incubated with a primary anti-BPIFA1 antibody at 4 °C over night (polyclonal sheep anti-mouse PLUNC—at 1:100; No. AF4274, Lot: ZLG011609A, R&D systems, Wiesbaden-Nordenstadt, Germany). Incubation with an immunopurified, irrelevant sheep antibody at a similar dilution served as negative control. The slides were incubated with horseradish peroxidase (HRP)-conjugated secondary rabbit anti-sheep IgG (1:200; No. P0163, Lot: 00001552, Dako, Hamburg, Germany). Diaminobenzidine (DAB) was used as substrate for color development. The slides were counterstained with hematoxylin, dehydrated through graded ethanol, cleared in xylene and coverslipped. BPIFA1-positive cells (%) per 100 µm basement membrane were determined in the distal trachea.

Immunohistochemical double labeling of BPIFA1 and macrophage-marker CD68 was performed using the H_2_O-elution method as described previously (Dietert et al. 2015). Slides were prepared as described above and incubated with anti-BPIFA1 antibody (polyclonal sheep anti-mouse PLUNC at 1:100) at 4 °C overnight. After incubation with HRP-conjugated secondary rabbit anti-sheep IgG (1:200), DAB was used as substrate for color development. Slides were washed in heated, deionized water (750 ml microwaved at 600 W for 10 min) to eliminate remaining unbound primary antibodies with a consecutive rinse in water at 4 °C for 5 min. Following incubation with the anti-CD68 antibody (polyclonal rabbit anti-mouse CD68 at 1:500; No. ab125212, Lot: GR77386-34, abcam, Cambridge; UK) at 4 °C over night and with the alkaline phosphatase (AP)-conjugated secondary goat anti-rabbit IgG (AP-1000 at 1:500; No. AP-1000, Lot: T1116, Vector, Burlingame, CA), triaminotritolyl-methanechloride (neufuchsin; NF) was used as substrate for color development. Alternatively, slides were incubated with an irrelevant immunopurified mouse or sheep antibody as negative controls. To ensure specific binding of the secondary HRP- or AP-conjugated antibodies with the BPIFA1- or CD68-specific primary antibodies, respectively, slides were incubated with only one primary but with both secondary antibodies. Finally, slides were counterstained with hematoxylin, dehydrated, cleared and coverslipped.

Images were acquired using an Olympus BX41 microscope (Olympus Deutschland GmbH, Hamburg, Germany) with an Olympus objective lens (PlanC N, 60x/0.80, ∞/017/FN22), the Olympus DP80 Dual CCD camera with 12.5 megapixel, and Olympus acquisition software CellSens Standard V1 (Version 1.13, iso 200 detector gain, 1360 × 1024 pixel resolution, 72 dpi, 24 image bit depth with automatic resolution time). Image processing in terms of white balance and compilation was performed via Adobe Photoshop CS5 Extended Version (Version 12.0.4).

### Microarray analyses

mRNA samples of alveolar macrophages stimulated with CLCA1-CM or incubated with pcDNA-CM as negative controls (*n* = 3) were subjected to mRNA gene expression profiling via microarray analysis (Hummingbird Diagnostics GmbH, Heidelberg, Germany). Sufficient quality of RNA samples was assessed with the Agilent 2100 Bioanalyzer and Nano RNA Kit according to the manufacturer’s instructions (Agilent Technologies, Santa Clara, USA). RNA was spectrophotometrically quantified using the Nanodrop 1000 instrument (Thermo Scientific, Waltham, USA). mRNA was labeled using Agilent’s Low Input Quick Amp Labeling Kit. After rotating hybridization for 16 h at 65 °C, slides were washed and scanned on Agilent’s SureScan Microarray Scanner. Image files from the scanner were transformed to raw data using Feature Extraction Software.

### Statistics

Data are expressed as mean ± standard error of the mean (SEM), statistically analyzed by the Mann–Whitney *U* test and graphically illustrated using GraphPad PRISM 6 (GraphPad Software Inc., La Jolla, USA). *p* < 0.05 was considered significant. RT-qPCR data are displayed as single value fold change. Dotted lines indicate fold changes of 0.5 and 2 as limits for valid statement of lowered or elevated expressions, respectively. The ΔΔCt (Ct, cycle threshold) method allowed for relative expression level quantification and group comparison and was based on data obtained from macrophages incubated with pcDNA-CM or PBS-treated WT control animals (fold change = 1). For protein expression analysis via CBA and multiplex assay, dotted lines indicate the detection limit of 10 pg/ml. For mRNA gene expression profiling, parametric *t* test and empirical Bayes statistics were applied. For both tests, an adjusted *p* value of < 0.05 was considered significant and a log_2_ of estimated fold change (log_2_ FC) value cutoffs of 1 and − 1 were considered as limits for valid statement of lowered or elevated expressions, respectively.

## Results

### CLCA1 stimulates cytokine expression in murine alveolar macrophages

Recently, human CLCA1− but not control-CM was shown to induce a pro-inflammatory response in a human monocyte cell line and also in primary porcine alveolar macrophages in vitro (Ching et al. 2013). Based on several previously reported differences of CLCA1 function between human and mouse models, we tested for CLCA1−-mediated alveolar macrophage activation in a solely murine context.

**Fig. 2:**
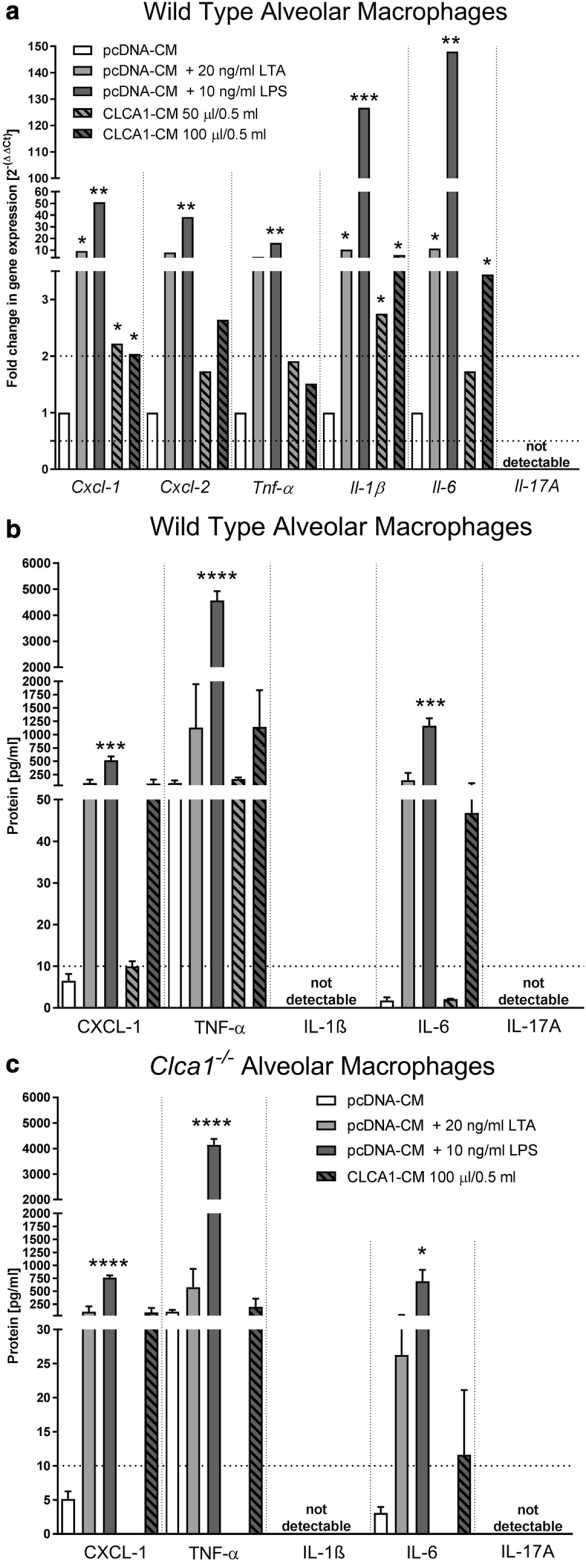
CLCA1 induced inflammatory cytokine gene expression in alveolar macrophages. Alveolar macrophages isolated from BALF were incubated with CLCA1-CM, LPS or LTA in pcDNA-CM as positive controls or pcDNA-CM alone as negative control for 24 h. **a** mRNA expression levels of inflammatory cytokines were quantified via RT-qPCR. Dotted lines indicate fold changes of 0.5 and 2 as limits for valid statement of lowered or elevated parameters, respectively. *Ct* cycle threshold. *n* = 3–5 per group. **b, c** Protein expression levels of inflammatory cytokines in cell culture supernatants from **b** wild-type and **c** *Clca1*^−/−^ macrophages were quantified by CBA or cytokine protein multiplex assay. *n* = 3–5 per group.*p < 0.05, **p < 0.01, ***p < 0.001, and ****p < 0.0001 vs. pcDNA controls

**Fig. 3:**
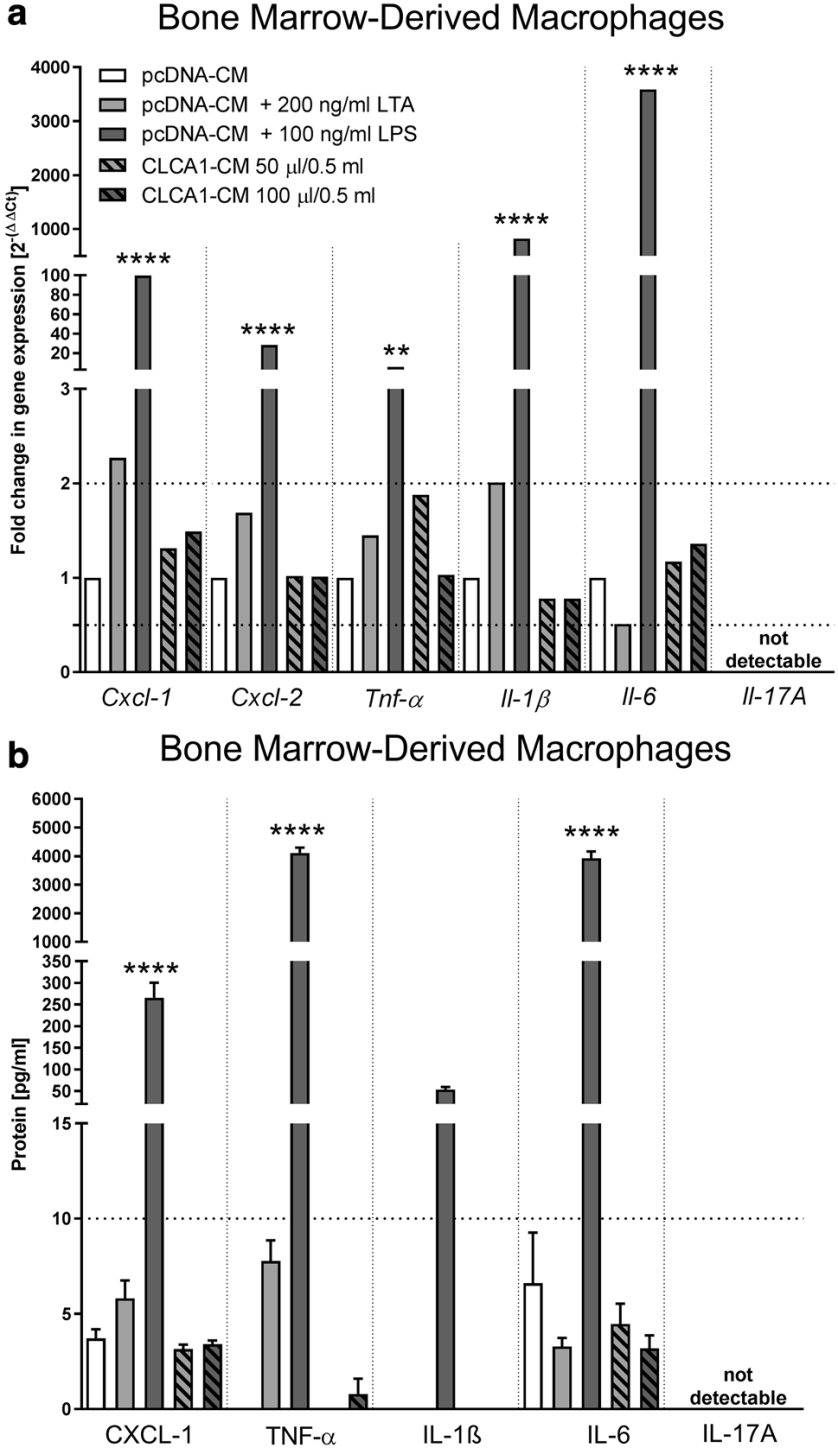
CLCA1 failed to induce cytokine expression in BMDM. After stimulation of macrophage cell cultures with CLCA1-CM, LPS or LTA in pcDNA-CM as positive controls or pcDNA-CM alone as negative control for 24 h, **a** mRNA expression levels of inflammatory cytokines were determined by RT-qPCR. Dotted lines indicate fold changes of 0.5 and 2 as limits for valid statement of lowered or elevated parameters, respectively. *Ct* cycle threshold. *n* = 3–5 per group. **b** Protein expression levels of inflammatory cytokines in macrophage cell culture supernatants were quantified by CBA or cytokine protein multiplex assay. *n* = 3–5 per group. **p < 0.01, and ****p < 0.0001 vs. pcDNA controls

Stimulation of alveolar macrophages from WT mice with CLCA1-CM resulted in upregulation of *Cxcl-1, Cxcl-2, Il-1β* and *Il-6* mRNA levels when compared to incubation with supernatant from pcDNA-transfected HEK293 cells (Fig. 2a). When 50 or 100 µl of CLCA1-CM were used, *Cxcl-2, Il-1β* and *Il-6* appeared to be upregulated in a dose-dependent fashion. No changes in *Tnf-α* expression were detected for both 50 or 100 µl of CLCA1-CM used. *Il-17A* mRNA was undetectable under the conditions tested. Stimulation with the known macrophage activators LTA and LPS resulted in marked increases of expression for all cytokines measured, except for *Il-17A*. On the protein level, WT alveolar macrophages also showed a dose-dependent CLCA1-mediated activation resulting in increased expression of CXCL-1, TNF-α, and IL-6 after incubation with 100 µl of CLCA1-CM (Fig. 2b). Here, cytokine expression levels were similar to those after stimulation with 20 ng/ml LTA. However, incubation with 100 µl of CLCA1-CM failed to induce any of the cytokine expression levels beyond those observed after incubation with supernatant from pcDNA-transfected HEK293 cells. Lack of extracellular IL-1β protein detection in alveolar macrophages may be explained by differences in LPS concentrations used for stimulation. It is well established that IL-1β is released extracellularly after stimulation solely with 0.5–1 µg/ml LPS (Beuscher et al. 1990). Alveolar macrophages, however, were stimulated with solely 10 ng/ml which represents a 50-fold lower concentration than that necessary for IL-1β release and explains the lack of extracellular IL-1β protein expression. In contrast, IL-1β protein was detected extracellularly in BMDM stimulated with 100 ng/ml LPS (Fig. 3b). This LPS concentration could not be used for alveolar macrophage activation due to cell toxicity (data not shown).

To test for an effect of CLCA1 deficiency on macrophage activation capacity, we also used alveolar macrophages from *Clca1*^−/−^ mice. To this end, the response patterns of alveolar macrophages derived from *Clca1*^−/−^ mice were compared to the responses observed in alveolar macrophages from WT mice using 100 µl of CLCA1-CM. The data yielded virtually identical results in macrophages from *Clca1*^−/−^ mice (Fig. 2c), suggesting that previous CLCA1 contact does not play a role during this macrophage activation. The levels of cytokine induction of CXCL-1 and TNF-α post-CLCA1-CM stimulation were comparable to those after LTA and pcDNA-CM stimulation, respectively. IL-1β and IL-17A were not expressed on the protein level in WT or in *Clca1*^−/−^ alveolar macrophages.

### CLCA1 does not activate BMDM

In contrast to alveolar macrophages, BMDM incubated with CLCA1-CM failed to increase expression levels of any of the cytokines tested on the mRNA (Fig. 3a) or protein level (Fig. 3b). In contrast to LPS but similar to CLCA1-CM, LTA failed to activate cytokine expression in BMDM. Again, IL-17A was not expressed on the mRNA and protein levels.

### Global gene expression analyses identified additional CLCA1-regulated genes involved in inflammation

A global gene expression analysis identified other genes that were differentially regulated upon stimulation of alveolar macrophages with 100 µl of CLCA1-CM compared to incubation with CM from pcDNA-transfected HEK293 cells. Virtually all of these genes are involved in early immune functions (Table 1). The most strongly up-regulated gene was *Ccl5*, also known as RANTES, with a log_2_FC of − 2.71 (Table 1). On the other hand, *Bpifa1* was the most prominently down-regulated gene with a log_2_FC of 2.18 (Table 1).

**Table 1:** Top 10 CLCA1-regulated coding genes as identified by global gene expression analysis. RNA gene expression profiling of CLCA1-CM-versus pcDNA-CM-stimulated WT alveolar macrophages was performed via microarray analysis and identified other genes regulated by CLCA1 which play a role in early immune responses

Gene name	Description	log FC	Biological relevance
*Ccl5*	Chemokine (C-C motif) ligand 5 (Ccl5)	− 2.71	Plays an active role in leukocyte recruitment to sites of inflammation, including T cells, macrophages, eosinophils, and basophils (Aldinucci and Colombatti 2014)
*Emr4*	EGF-like module containing, mucin-like, hormone receptor-like sequence 4 (Emr4)	− 2.26	Predominantly expressed on resident macrophages, expression is up-regulated following macrophage activation (Stacey et al. 2002)
*Bpifa1*	BPI fold containing family A, member 1 (Bpifa1)	2.18	Abundantly secreted in the respiratory tract, antimicrobial- and anti-biofilm properties, regulates mucociliary clearance and downstream chemokine CCL24 (eotaxin-2) expression (Britto and Cohn 2015)
*Ccr7*	Chemokine (C-C motif) receptor 7 (Ccr7)	− 2.15	Expressed in lymphoid tissues, activates B and T lymphocytes, controls the migration of memory T cells to inflamed tissues (NCBI—C–C motif chemokine receptor 7 2016), regulates T cell trafficking and compartmentalization within secondary lymphoid organs (Sharma et al. 2015)
*Il-1β*	Interleukin 1 beta (Il-1β)	− 2.13	Produced by activated macrophages, mediator of the inflammatory response (NCBI—Interleukin-1 beta 2016a), involved in cell proliferation, differentiation, and apoptosis (NCBI—Interleukin-1 beta 2016b)
*Aoah*	Acyloxyacyl hydrolase (Aoah)	− 2.13	Enzyme that catalyzes the hydrolysis of acyl chains from bacterial LPS, modulates host inflammatory response to Gram-negative bacteria (NCBI—acyloxyacyl hydrolase 2016)
*Ly6i*	Lymphocyte antigen 6 complex, locus I (Ly6i)	− 2.12	Takes part in differentiation of various hematopoietic lineages, highly present on immature granulocytes and monocytes, could function in a common pathway for T and B lymphocyte development, useful in characterizing different stages of T lymphocyte development and activation (Pflugh et al. 2000)
*Ly6c1*	Lymphocyte antigen 6 complex, locus C1 (Ly6c1)	− 2.12	Murine cell surface protein, identified on specific subsets of resting lymphocytes (Rock et al. 1989), pro-inflammatory cytokines transiently up-regulate B cell Ly-6C expression (Schlueter et al. 2002)
*H2-M2*	Histocompatibility 2, M region locus 2 (H2-M2)	− 2.11	Surface-expressed MHC class I molecule with a function still to be elucidated (Moore et al. 2004)
*Eif2s3y*	Eukaryotic translation initiation factor 2, subunit 3, structural gene Y-linked (Eif2s3y)	2.04	Subunit of eukaryotic initiation factor 2 (eIF2), involved in the early steps of protein synthesis (Genetics Home Reference—EIF2S3 gene 2016)

According to the applied statistical tests using the adjusted *p* value, significantly deregulated transcripts could not be identified. Log fold changes of > 1 and < − 1 as limit for valid statement of lowered and elevated parameters for CLCA1-CM-stimulated alveolar macrophages vs. pcDNA-CM controls. *n* = 3 per group. Note that a negative value indicates up-regulation, a positive value down-regulation

**Fig. 4:**
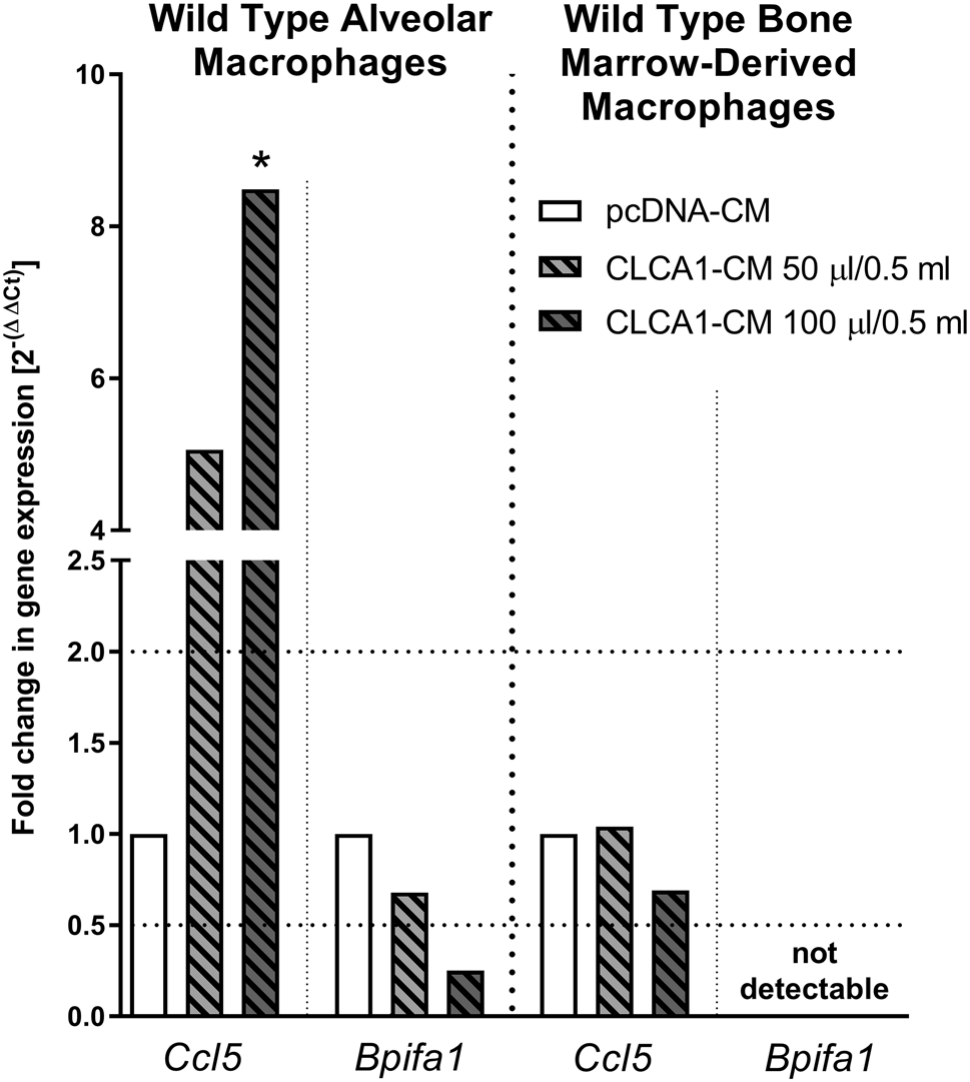
CLCA1-dependent regulation of *Ccl5* and *Bpifa1* was verified by RT-qPCR Representatively, the two most regulated genes, *Ccl5* and *Bpifa1*, were analyzed after 24 h of incubation of alveolar and bone marrow-derived macrophages with CLCA1-CM compared to pcDNA-CM via RT-qPCR as a methodical gold standard. Dotted lines indicate fold changes of 0.5 and 2 as limits for valid statement of lowered or elevated parameters, respectively. *Ct* cycle threshold. *p < 0.05 vs. pcDNA controls. *n* = 3–5 per group

These data were verified by RT-qPCR analyses confirming that *Ccl5* was dose-dependently up-regulated whereas *Bpifa1* mRNA was dose-dependently down-regulated in alveolar macrophages upon incubation with CLCA1-CM (Fig. 4). In contrast to alveolar macrophages, however, RT-qPCR data from BMDM failed to identify changes in *Ccl5* expression after both CLCA1-CM stimulation and incubation with CM from pcDNA-transfected HEK 293 cells whilst *Bpifa1* expression was not detected (Fig. 4).

**Fig. 5:**
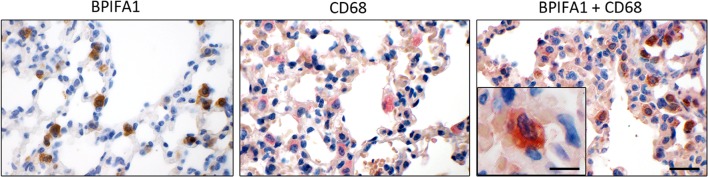
BPIFA1 is expressed in murine alveolar macrophages. Immunohistochemistry of murine lung sections with anti-BPIFA1 antibody (DAB, brown, left image), anti-CD68 antibody (NF, red, middle image), and double staining (right image). Bar 50 µm; bar inset 20 µm

In contrast to our microarray and RT-qPCR data clearly showing *Bpifa1* expression in alveolar macrophages, BPIFA1 has to date not been reported to be expressed in this cell type. To verify its expression on the protein level, immunohistochemical single and double stainings for the BPIFA1 protein and the macrophage-marker CD68 were performed on murine lungs which were obtained from a previous *S. aureus* infection study on *Clca1*^−/−^ and WT mice with PBS-treated controls. BPIFA1 signals were clearly present in the cytoplasm of CD68-expressing alveolar macrophages (Fig. 5), independently of genotype or the stimulus used.

### BPIFA1 was differentially expressed in WT mice but not in *Clca1*^−/−^ mice in a model of acute *S. aureus* pneumonia

**Fig. 6:**
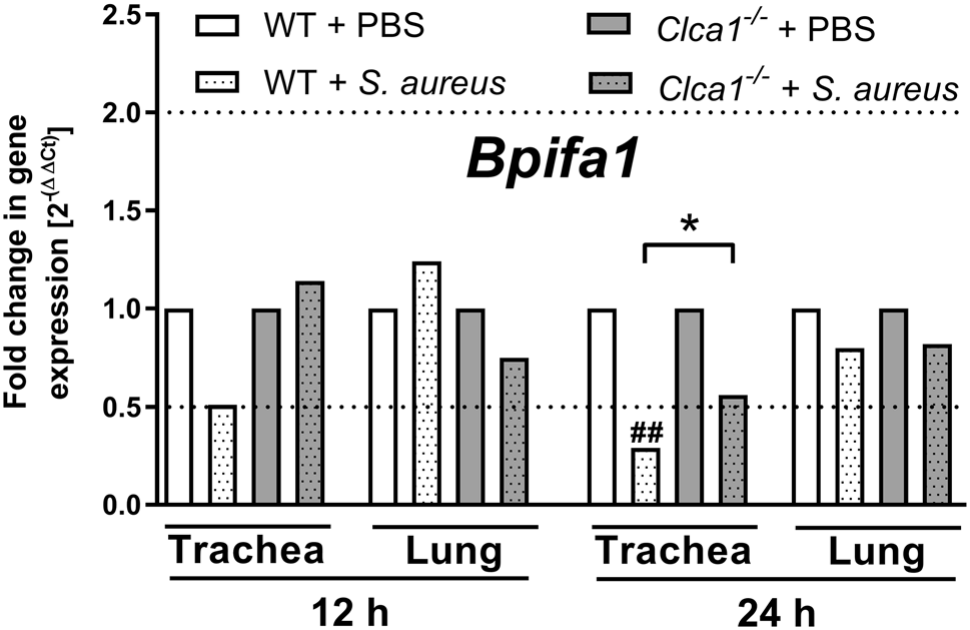
*Bpifa1* expression was decreased in murine WT but not *Clca1*^−/−^ tracheal tissue in *S. aureus* pneumonia. *Bpifa1* expression was determined via qRT-PCR in *Clca1*^−/−^ and WT tracheal and lung tissue from a murine model of acute pneumonia 12 and 24 h post-infection with *S. aureus*. Dotted lines indicate fold changes of 0.5 and 2 as limits for valid statement of lowered or elevated parameters, respectively. *Ct* cycle threshold. *p < 0.05 vs. WT, ^##^*p* < 0.01 vs. PBS controls. *n* = 8–11 per group

We further tested *Bpifa1* expression in a murine model of acute *S. aureus* pneumonia in *Clca1*^−/−^ versus WT mice to correlate CLCA1 and BPIFA1 not only in vitro but also in vivo. *Bpifa1* mRNA was significantly decreased in the trachea of *S. aureus*-infected WT mice compared to PBS-treated controls 24 h post-infection (Fig. 6). In contrast, expression of *Bpifa1* was significantly increased in mice lacking CLCA1 compared to WT mice under conditions of *S. aureus* pneumonia in the trachea (Fig. 6). This confirmed the dependence of BPIFA1 regulation on the presence of CLCA1 in vivo. No effects on *Bpifa1* expression were observed in lung tissues at this point in time or in the trachea as well as in lung tissues at 12 h post-infection.

**Fig. 7:**
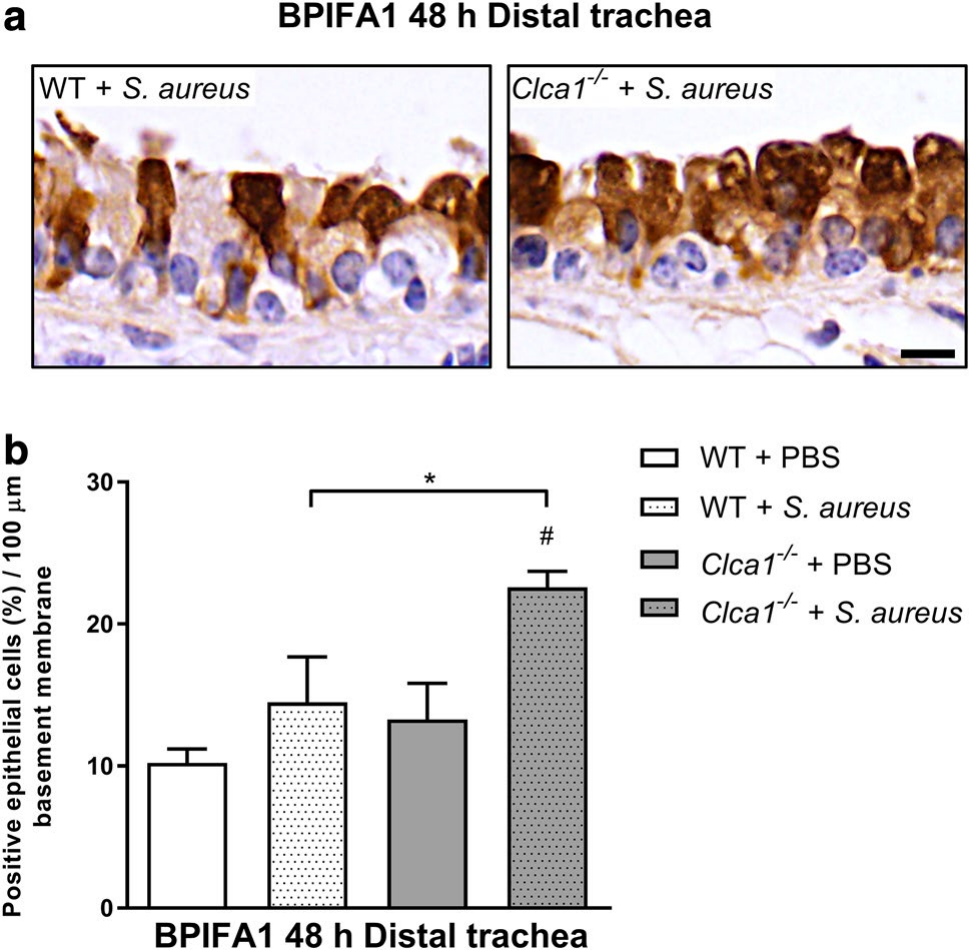
BPIFA1 is genotype dependently differentially expressed in the distal trachea in acute murine *S. aureus* pneumonia. BPIFA1 protein expression was examined in the distal trachea from PBS-treated mice and mice infected with *S. aureus* 48 h after infection. **a** BPIFA1 immunohistochemistry of representative sections and **b** percentage of BPIFA1-positive cells per 100 µm basement membrane of the distal trachea of *Clca1*^−/−^ mice compared to WT mice at 48 h post-infection. Bar 10 µm. *p < 0.05 vs. WT; ^#^*p* < 0.05 vs. PBS controls. *n* = 4 per group

Furthermore, BPIFA1 protein-expressing respiratory epithelial cells were quantified in the distal trachea in WT and *Clca1*^−/−^ mice with or without *S. aureus* infection, respectively (Fig. 7a). A significantly higher percentage of BPIFA1-positive respiratory epithelial cells per 100 µm basement membrane was observed at 48 h after infection in *Clca1*^−/−^ mice compared to WT mice in the distal trachea with 22.6 and 14.5% BPIFA1-expressing epithelial cells, respectively (*p* < 0.05; Fig. 7b). These results suggest that the lack of CLCA1 also affects expression of the BPIFA1 protein by respiratory epithelial cells.

## Discussion

It was recently shown that human CLCA1 triggered the release of selected inflammatory cytokines in a human airway macrophage–monocyte cell line and also in a primary porcine alveolar macrophage culture in vitro, opening a new functional perspective for CLCA1 (Ching et al. 2013). These data on human CLCA1 also yielded a first mechanistic approach toward the understanding of the phenotype of *Clca1*^−/−^ mice in a mouse pneumonia model (Dietert et al. 2014). However, due to considerable species-specific genomic differences within the CLCA family of genes and differences in postulated functions of CLCA1 in humans and mice, such as the induction of mucus cell metaplasia (Alevy et al. 2012; Patel et al. 2006) and the modulation of a non-CFTR mediated, calcium-dependent anion conductance (Mundhenk et al. 2012), the translation of functional observations between human and mouse CLCA proteins remains particularly challenging. To elucidate similarities and possible differences between human vs. murine CLCA1 related to this novel functional aspect, we tested CLCA1-mediated activation of macrophages in a solely murine context. We further extended our study to identify more downstream effector pathways in early immune functions, resulting in the discovery of BPIFA1 being regulated downstream of CLCA1, thereby widening the regulatory effects of CLCA1 in early immune responses.

As a first important result of this study, we established that mouse CLCA1 activates mouse alveolar macrophages similar to the recently described activation of human and pig macrophages by human CLCA1, pointing toward an evolutionarily conserved and thus probably substantial role of the secreted mucus protein CLCA1 in early airway inflammation. Specifically, CLCA1 activates alveolar macrophages, resulting in their expression of early pro-inflammatory cytokines, such as IL-1, IL-6 and TNF-α, as well as increased expression of leukotactic chemokines, including the human IL-8 homologues CXCL-1 and CXCL-2. As we used conditioned medium in this study, we attempted to exclude the possibility that secreted proteins from HEK 293 cells induced by CLCA1 are responsible for the effects. To this end, we analyzed murine and human cytokines via CBA in the supernatant of transfected HEK-293 cells, none of which was induced by CLCA1, as shown in Online Resource 4 (Electronic Supplementary Material). In the present study, murine CLCA1-conditioned HEK cell culture medium was employed instead of purified CLCA1 since the main limitation of any in vitro study using purified CLCA1 as an additive is the potential functionality compromise or alteration by CLCA1 purification processes. However, Ching et al. had shown that human CLCA1 purified by immunoprecipitation from CLCA1-cultured FBS-free medium and human CLCA1-cultured FBS-free medium itself yielded no difference in macrophage cytokine expression (Ching et al. 2013). Hence, that study had verified that macrophage activation is not dependent on constituents that might potentially be induced and secreted by human CLCA1. We therefore speculate that the effects seen in the present study are attributable to CLCA1.

The main phenotype of *Clca1*^−/−^ mice in a model of *S. aureus*-induced pneumonia consisted of reduced activation of CXCL-1 and IL-17A expression and decreased neutrophilic infiltration into the bronchoalveolar space (Dietert et al. 2014). Thus, the data obtained here convincingly explain reduced CXCL-1 activation and reduced leukocyte influx into the airspaces as an effect due to lack of stimulation of alveolar macrophages by CLCA1, which had also been proposed for the human CLCA1 in vitro (Ching et al. 2013). However, *Clca1*^−/−^ mice infected with *S. aureus* had also decreased activation of IL-17A, an effect obviously not mediated by alveolar macrophages (Fig. 2) which makes it plausible to assume that reduced IL-17A expression is due to reduced Th17-lymphocyte activation, with Th17-lymphocytes being the main source of IL-17A (Jin and Dong 2013; Song et al. 2008). It has been shown that macrophages, when stimulated with bacteria, could be the source of Il-17 which represents a necessary host defense against *Staphylococci* and *Citrobacter* (Cho et al. 2010; Ishigame et al. 2009; Jin and Dong 2013). Thus, we cannot exclude that using only the bacterially derived stimulants LPS or LTA and not bacteria themselves in our study accounts for the lack of Il-17A expression in vitro. On the other hand, since several of the CLCA1-induced chemokines in alveolar macrophages activate or attract lymphocytes, the reduced IL-17A expression seen in vivo can also be explained as an indirect sequela of lack of CLCA1 via reduced activation of T lymphocytes. Taken together, the results obtained here may fully explain all aspects of the phenotype of *Clca1*^−/−^ mice infected with *S. aureus* via reduced stimulation of alveolar macrophages by the lack of CLCA1.

It is known that priming during macrophage lineage maturation by certain colony-stimulating factors or interleukins may influence the macrophage phenotype and cytokine expression pattern (Vogel et al. 2014). Since CLCA1 has never been reported to be expressed by macrophages or other immune cells, we tested whether this alveolar macrophage phenotype underwent priming with CLCA1 using *Clca1*^−/−^ alveolar macrophages for stimulation. Virtually identical activation of alveolar macrophages isolated from *Clca1*^−/−^ mice compared to cells derived from WT mice suggested that this activation is completely independent of a previous macrophage contact with this protein and, furthermore, underscores the notion that CLCA1 expression by macrophages themselves does not play a role during this process.

To test for specificity of the activating effect for the proposed airway environment, ex vivo bone marrow-derived monocytes were subjected to a commonly used L929-conditioned macrophage differentiating medium, allowing to test for possible activation of non-airway macrophages by CLCA1. The lack of activation by CLCA1 indicates that non-airway macrophages may not serve as effector cells of this mucosa-associated secreted activator and that susceptibility to CLCA1 activation may be restricted to macrophages in mucosal environments where CLCA1 is normally present, such as the airways. Lung alveolar macrophages are indeed very different in nature from BMDM. Alveolar macrophages are derived from fetal precursors and self-renew in situ (Tarling et al. 1987; Yona et al. 2013). In addition, priming during macrophage lineage maturation by certain colony-stimulating factors or interleukins may influence the macrophage phenotype and cytokine expression pattern (Vogel et al. 2014). These aspects can contribute to the reaction pattern difference between alveolar and the comparatively naive BMDMs. To elucidate the latter aspect, future experiments should test whether tissue-specific macrophages in other mucosal linings, such as the intestine, female genital tract and salivary glands, in which CLCA1 is also secreted by mucin-producing cells (Leverkoehne and Gruber 2002), may also be activated by CLCA1.

Furthermore, stimulation of alveolar macrophages as well as the lack of BMDM activation after CLCA1-CM addition closely resembles the LTA-mediated activation pattern. Importantly, failure of BMDM activation by LTA, a TLR2 agonist (Schroder et al. 2003), is known since TLR2-mediated activation of naive BMDM is MyD88 dependent and requires cooperative signaling of both TLR2 and TLR4 (Papadopoulos et al. 2013) while LPS, the primary ligand of TLR4 (Takeda and Akira 2003), is sufficient for activation (Meng and Lowell 1997) as seen in our study. It remains to be established whether CLCA1 and LTA indeed share similar activation pathways.

In light of previously established species differences, especially regarding the functional differences of human and mouse CLCA1 concerning IL-13-induced airway mucus cell metaplasia (Alevy et al. 2012; Patel et al. 2006) and their modulation of a non-CFTR mediated calcium-dependent anion conductance (Mundhenk et al. 2012), it appears noteworthy that our data on mouse CLCA1 inducing the expression of several early pro-inflammatory cytokines and chemokines in mouse alveolar macrophages closely resemble the data observed in the human model (Ching et al. 2013). This points toward an evolutionarily conserved and thus probably substantial role of the secreted CLCA1 protein in early airway inflammation. Moreover, our observations add several further considerable contributions to the model of CLCA1 inducing early immune effects in airway macrophages.

As a second important outcome of this study, RNA expression profiling of CLCA1-CM-stimulated alveolar macrophages not only confirmed the activation of pro-inflammatory cytokines and chemoattractants of leukocytes in early inflammation but also identified further downstream effectors (Table 1). The most strongly down-regulated gene was the multifunctional 25 kDa secreted glycoprotein BPIFA1 which acts in immune defense mechanisms and liquid homeostasis in airway mucosal membranes (Britto and Cohn 2015). BPIFA1 regulates cytokine gene expression in macrophages, specifically causing a down-regulation of eosinophil chemokine CCL24 (Eotaxin-2) (Thaikoottathil and Chu 2011). Consistently, lungs of BPIFA1-deficient (*Bpifa1*^−/−^) mice have increased Eotaxin-2 expression (Thaikoottathil et al. 2012). Furthermore, it has recently been suggested that genetic polymorphisms in the *Bpifa1* gene are associated with the disease severity in CF (Liu et al. 2013b). BPIFA1 is thought to be primarily expressed by tracheal and bronchial epithelial cells in mice and humans, the latter with additional expression in submucosal glands (Campos et al. 2004; Di et al. 2003) which are largely absent from mice. However, BPIFA1 expression has never been reported in hematopoietic cells other than neutrophils (Britto and Cohn 2015) which is controversially discussed. Specifically, co-localization of BPIFA1 with macrophages/monocytes in human CF lungs has failed to date (Bingle et al. 2007). Our immunohistochemical data not only confirm that airway epithelial cells represent the majority of BPIFA1-expressing cells but also clearly establish that mouse airway macrophages express this protein and that its BPIFA1 expression is obviously modulated by CLCA1. Furthermore, when we re-examined tissues from our earlier infection experiment (Dietert et al. 2014) in terms of altered expression of BPIFA1 in *Clca1*^−/−^ mice, we were able to confirm the effect seen ex vivo to be true for the in vivo scenario of *S. aureus*-induced pneumonia in that *Bpifa1* mRNA was less reduced in *Clca1*^−/−^ mice at 24 h post-infection in the trachea (Fig. 6). Moreover, *Clca1*^−/−^ mice infected with *S. aureus* had significantly higher numbers of BPIFA1-expressing respiratory epithelial cells 48 h after infection when compared to WT mice (Fig. 7), arguing that modulation of BPIFA1 expression by respiratory epithelial cells may also be dependent on CLCA1. Since the only BPIFA1-expressing cells in the trachea were identified immunohistochemically as respiratory epithelial cells, it appears plausible to assume that altered BPIFA1 regulation in *Clca1*^−/−^ mice is based on this specific cell type. However, the combination of our results using ex vivo alveolar murine macrophages stimulated with CLCA1 (Table 1; Fig. 4) and our immunohistochemical quantification of BPIFA1-expressing epithelial cells (Fig. 7) suggests that both cell types may be involved in CLCA1 mediation of BPIFA1 expression. It will be interesting to explore in greater detail the mechanisms of altered BPIFA1 modulation in *Clca1*^−/−^ mice, and whether there may even be a direct regulatory role for the soluble mucus protein CLCA1 on airway epithelial cells.

When comparing certain cytokine expression levels in infectious models of *Bpifa1*-knockout (*Bpifa1*^−/−^) and *Clca1*^−/−^ mice, we found interesting parallels. In a murine *Bpifa1*^−/−^ model of pulmonary *Pseudomonas* (*P*.) *aeruginosa* infection, *Bpifa1*^−/−^ mice had significantly increased expression of, amongst other cytokines, *Cxcl-1* and *-2* in lung tissue with subsequently increased total inflammatory cell number and neutrophils in BALF and enhanced pneumonia (Liu et al. 2013b). This phenotype might be secondary to the lack of BPIFA1 as an antimicrobial protein with increased bacterial burden. However, since BPIFA1 is capable of regulating cytokine expression in alveolar macrophages (Thaikoottathil et al. 2012), it is conceivable that some aspects of the observed phenotype may be due to the lack of BPIFA1 as a cytokine regulator. Virtually identical to the *Bpifa1*^−/−^ mice, the expression of CXCL-1 and -2 was also decreased in the *Clca1*^−/−^ mouse model of acute *S. aureus* pneumonia, with consequently decreased numbers of airway infiltrating leukocytes, specifically of neutrophils (Dietert et al. 2014).

The discovery of a signaling link between CLCA1 and BPIFA1 also sheds new light on the alleged role of CLCA1 in anion conductance and liquid homeostasis, which originally gave rise to the designation of this gene family as “chloride channel regulators, calcium-activated” (Patel et al. 2009). Very recently, conspicuous parallels concerning interspecies diversities and similar microenvironment expression patterns were found between CLCA1 and BPIFA1 (Mundhenk et al. 2018) which underlines potential overlapping effects of CLCA1 and BPIFA1. In light of several previous and partially contradictory reports on human and mouse CLCA1 and other CLCA proteins modulating anion conductance in epithelial cells (Gruber et al. 1998; Mundhenk et al. 2012), it is tempting to speculate that this effect may also involve BPIFA1, which has been well established as a modifier of airway liquid homeostasis, including modulation of the epithelial sodium channel (ENaC) (Gaillard et al. 2010; Garland et al. 2013; Hobbs et al. 2013).

## Electronic supplementary material

Below is the link to the electronic supplementary material.

Supplementary material 1 (PDF 487 KB)
