# Supplementary material for: Soluble mucus component CLCA1 modulates expression of leukotactic cytokines and BPIFA1 in murine alveolar macrophages but not in bone marrow-derived macrophages
Source: Histochem Cell Biol. 2018 Apr 2;149(6):619–33. doi: 10.1007/s00418-018-1664-y (PMC5999134; doi:10.1007/s00418-018-1664-y)
Supplement: Supplementary file 1 — Supplementary material 1 (PDF 487 KB) [file 418_2018_1664_MOESM1_ESM.pdf]

Soluble Mucus Component CLCA1 Modulates Expression of Leukotactic Cytokines and BPIFA1 in Murine Alveolar Macrophages But Not in Bone Marrow-Derived Macrophages

Journal: Histochemistry and Cell Biology

Authors: Nancy A. Erickson, Kristina Dietert, Jana Enders, Rainer Glauben, Geraldine Nouailles, Achim D. Gruber, and Lars Mundhenk

Corresponding author: Lars Mundhenk, Department of Veterinary Pathology, Freie Universität Berlin, Robert-von-Ostertag-Strasse 15, 14163 Berlin

lars.mundhenk@fu-berlin.de

Online Resource 1: Determination of CLCA1 protein in the conditioned medium

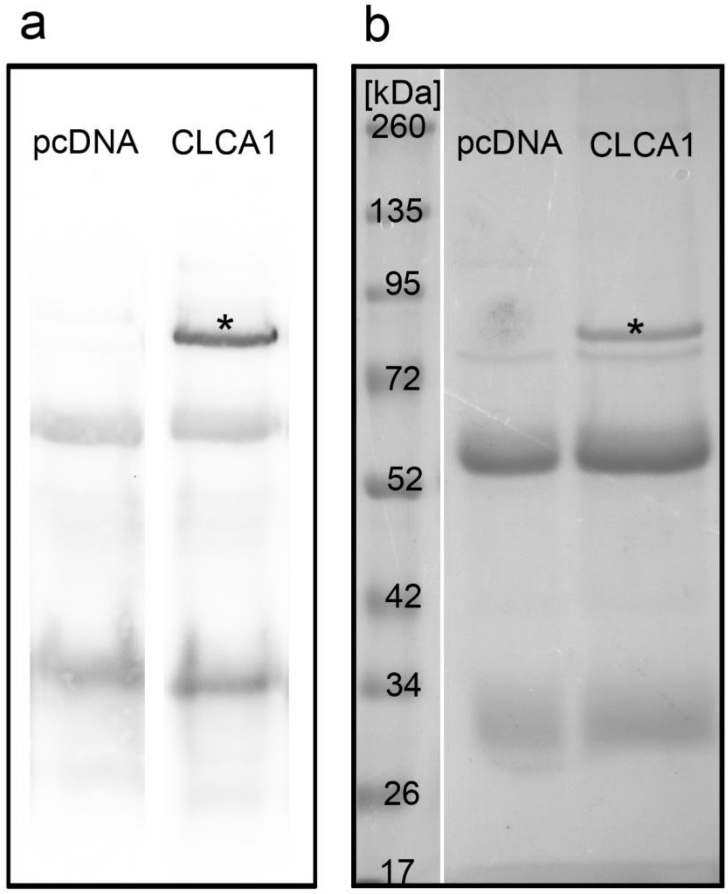

Prior to macromolecule concentration via Vivaspin, 500 µl of supernatant of CLCA1- or pc-transfected HEK 293 cells were subjected to immunoprecipation using the α-mCLCA3-C-1p antibody. Immunoprecipitates were separated by SDS-PAGE. Representative images of (a) immunoblot analysis of the immunoprecipitates using α-p3b2 identifying specifically the approximately 75 kDa amino-terminal cleavage product of CLCA1 (*) in the supernatant of CLCA1-transfected cells and (b) the 75 kDa protein band (*) of the immunoprecipitated CLCA1 protein compared to equally processed pcDNA-CM on a Coomassie-stained SDS-PAGE.

Online Resource 2: Experimental Setup*

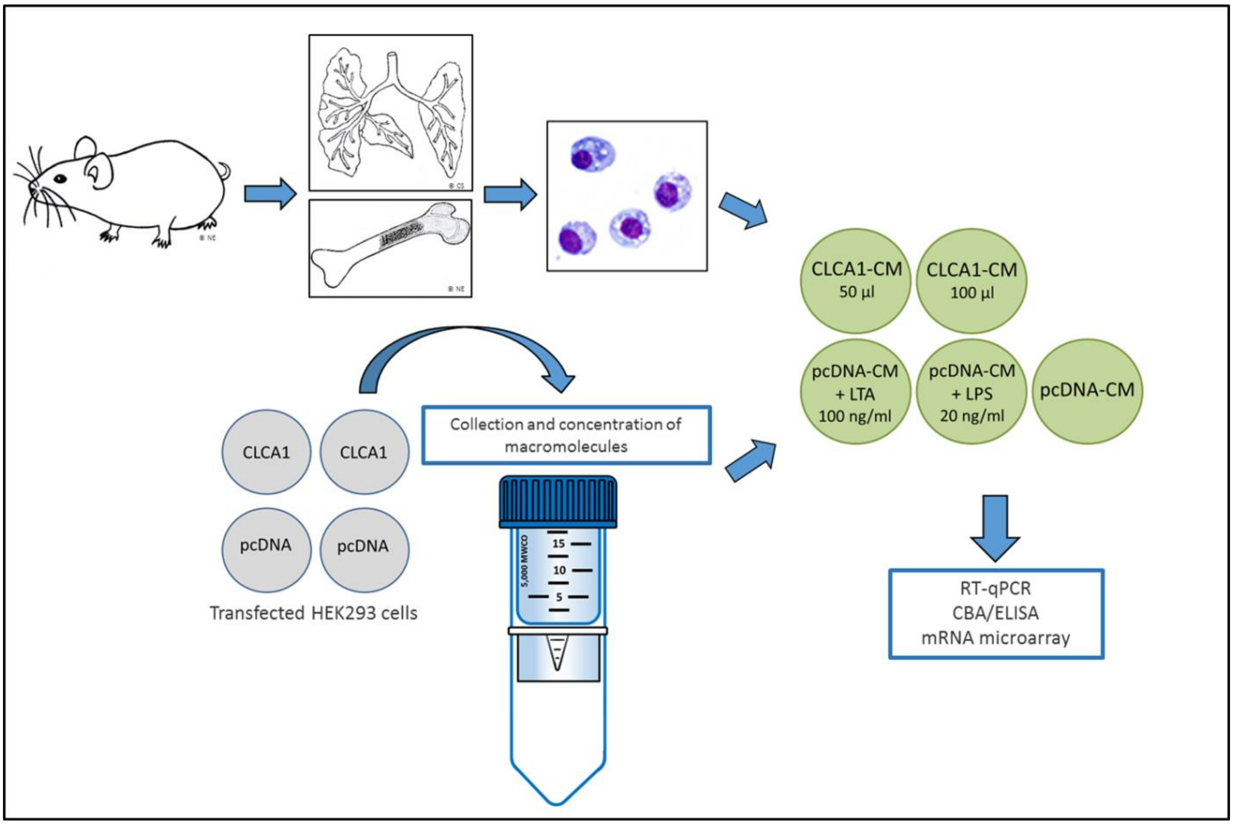

*Artwork created using Microsoft PowerPoint 2010

Online Resource 3: Quantitative RT-PCR – primer and probe sequences

Gene	Genbank Accession No.	Oligonucleotide Sequences (5'-3')	Reference
Cxcl-1<br>*(KC)*	NM_008176.3	Primer (upstream) GATGCTAAAAGGTGTCCCCA<br>Primer (downstream)AGACTGCTCTGATGGCACCT<br>TaqMan Probe FAM-GTCAGAAGCCAGCGTTCAC-BHQ	(Dietert et al. 2014)
Cxcl-2<br>*(MIP-2α)*	NM_009140.2	Primer (upstream) CTGAACAAAGGCAAGGCTAACT<br>Primer (downstream)ACCTGGAAAGGAGGAGCCT<br>TaqMan Probe FAM-CTTTGGTTCTTCCGTTGAGG-BHQ	(Dietert et al. 2014)
Il-1β	NM_008361.3	Primer (upstream) CAACCAACAAGTGATATTCTCCATG<br>Primer (downstream)GATCCACACTCTCCAGCTGCA<br>TaqMan Probe FAM-CTGTGTAATGAAAGACGGCACACCCACC-BHQ	(Giulietti et al. 2001)
Il-6	NM_031168.1	Primer (upstream) CCGGAGAGGAGACTTCACAGA<br>Primer (downstream)AGAATTGCCATTGCACAACTCTT<br>TaqMan Probe FAM-ACCACTTCACAAGTCGGAGGCTTAATTACA-BHQ	(Bloks 2009)
Il-17	NM_010552	Primer (upstream) GCTCCAGAAGGCCCTCAGA<br>Primer (downstream)CTCTCCACCGCAATGAAGACCCTGA<br>TaqMan Probe FAM-AGCTTTCCCTCCGCATTGA-BHQ	(Giulietti et al. 2001)
Tnfα	NM_013693.3	Primer (upstream) CATCTTCTCAAAATTCGAGTGACAA<br>Primer (downstream)TGGGAGTAGACAAGGTACAACCC<br>TaqMan Probe FAM-CACGTCGTAGCAAACCACCAAGTGGA-BHQ*	(Innamorato et al. 2008)
Bpifa1	NM_011126.3	Primer (upstream) TGGGATTCTCAGCGGTTTGGATGT<br>Primer (downstream)TCAGCCAAGATAGCCTTCCTTCCT<br>TaqMan Probe FAM-CACCCTGGTGCACAACATTGCTGAAT-BHQ	(Liu et al. 2013)
Ccl5	NM_013653.3	Primer (upstream) AGATCTCTGCAGCTGCCCTCA<br>Primer (downstream)GGAGCACTTGCTGCTGGTGTAG<br>TaqMan Probe FAM-CTCGTGCCCACGTCAAGGAGTAT-BHQ*	(Ishida et al. 2012)*
Ef-1a	NM_010106.2	Primer (upstream) AAAAACGACCCACCAATGG<br>Primer (downstream)GGCCTGGATGGTTCAGGATA<br>TaqMan Probe FAM-AGCAGCTGGCTTCACGCTCAGGTG -BHQ	(Braun et al. 2010)
B2m	NM_009735.3	Primer (upstream) ATTCACCCCCACTGAGACTGA<br>Primer (downstream)CTCGATCCCAGTAGACGGTC<br>TaqMan Probe FAM-TGCAGAGTTAAGCATGACAGTATGGCCG -BHQ	(Norris et al. 2000)
Gapdh	NM_008084.2	Primer (upstream) TCACCACCATGGAGAAGG<br>Primer (downstream)GCTAAGCAGTTGGTGGTGCA<br>TaqMan Probe FAM-ATGCCCCCATGTTTGTGATGGGTGT-BHQ	(Giulietti et al. 2001)

*Probe designed by authors

References

Bloks V (2009) RTPrimer - DB ID 8140. RTPrimerDB: the Real-Time PCR primer and probe database. http://www.rtprimerdb.org/assay_report.php?assay_id=8140. Accessed 21 Oct 2016

Braun J, Mundhenk L, Range F, Gruber AD (2010) Quantitative expression analyses of candidates for alternative anion conductance in cystic fibrosis mouse models Journal of Cystic Fibrosis: Official Journal of the European Cystic Fibrosis Society 9:351-364 doi:10.1016/j.jcf.2010.06.003

Dietert K, Reppe K, Mundhenk L, Witzenrath M, Gruber AD (2014) mCLCA3 modulates IL-17 and CXCL-1 induction and leukocyte recruitment in murine Staphylococcus aureus pneumonia PloS One 9:e102606 doi:10.1371/journal.pone.0102606

Giulietti A, Overbergh L, Valckx D, Decallonne B, Bouillon R, Mathieu C (2001) An overview of real-time quantitative PCR: applications to quantify cytokine gene expression Methods 25:386-401 doi:10.1006/meth.2001.1261

Innamorato NG, Rojo AI, Garcia-Yague AJ, Yamamoto M, de Ceballos ML, Cuadrado A (2008) The transcription factor Nrf2 is a therapeutic target against brain inflammation Journal of immunology (Baltimore, Md : 1950) 181:680-689

Ishida Y, Kimura A, Kuninaka Y, Inui M, Matsushima K, Mukaida N, Kondo T (2012) Pivotal role of the CCL5/CCR5 interaction for recruitment of endothelial progenitor cells in mouse wound healing The Journal of Clinical Investigation 122:711-721 doi:10.1172/jci43027

Liu MJ et al. (2013) ZIP8 regulates host defense through zinc-mediated inhibition of NF-kappaB Cell Reports 3:386-400 doi:10.1016/j.celrep.2013.01.009

Norris MD, Burkhart CA, Marshall GM, Weiss WA, Haber M (2000) Expression of N-myc and MRP genes and their relationship to N-myc gene dosage and tumor formation in a murine neuroblastoma model Medical and Pediatric Oncology 35:585-589

Online Resource 4: CBA Analysis of CLCA1-CM

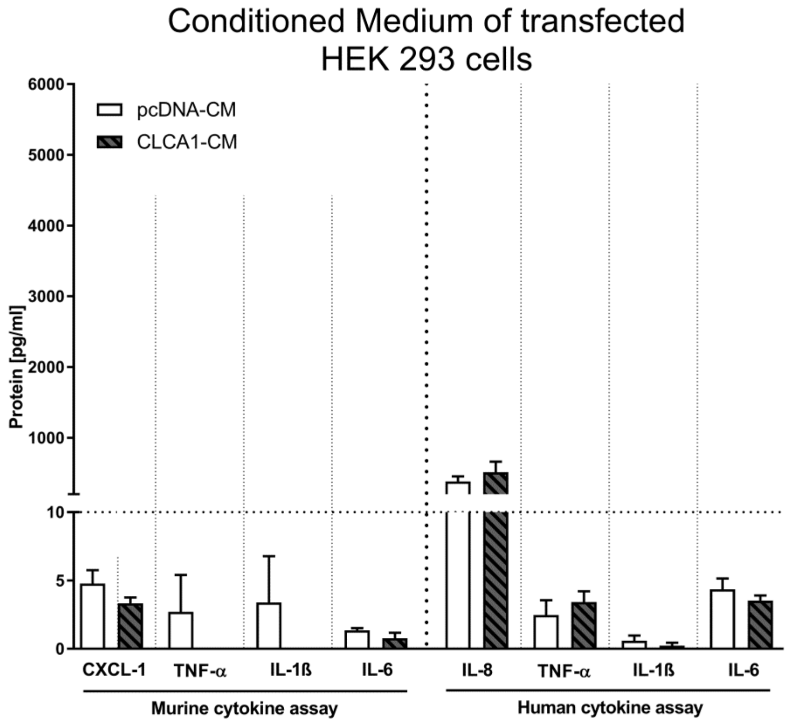

To exclude introduction of cytokines with the CM of CLCA1 transfected HEK293 cells, the supernatants of these cells were analyzed for cytokine induction via murine and human CBA assay. The human IL-8 was the only cytokine measured in the supernatant above the valid detection limit of 10 pg/ml (dotted line), however, without any differences between CLCA1- and pcDNA-transfected cells. Data are expressed as mean ± standard error of the mean (SEM), statistically analyzed by the Mann-Whitney-U test and graphically illustrated using GraphPad PRISM 6. $P < 0.05$ was considered significant.
